# Associations of social isolation, social participation, and loneliness with frailty in older adults in Singapore: a panel data analysis

**DOI:** 10.1186/s12877-021-02745-2

**Published:** 2022-01-06

**Authors:** Lixia Ge, Chun Wei Yap, Bee Hoon Heng

**Affiliations:** Health Services & Outcomes Research, National Healthcare Group, Singapore, Singapore

**Keywords:** Frailty, Social isolation, Social participation, Loneliness, Older adults

## Abstract

**Background:**

There is a shortage of research evidence about how social isolation, social participation, and loneliness were longitudinally associated with frailty. This study was to 1) examine the associations of social isolation, social participation, and loneliness with level of frailty among community-dwelling older adults using panel data, and 2) explore the moderating effect of gender on the association of social isolation, social participation and loneliness with frailty.

**Methods:**

The study included 606 participants aged 60 years and above from the longitudinal Population Health Index Survey conducted in Singapore. At each timepoint, level of frailty was determined using the Clinical Frailty Scale. Social isolation was assessed by the Lubben Social Network Scale-6, and loneliness was assessed using the three-item UCLA Loneliness Scale. Fixed-effects ordinal logistic regressions were conducted with level of frailty as the dependent variable and social isolation and loneliness as the independent variables, adjusting for time-varying socio-demographic, lifestyle, and health-related factors.

**Results:**

Increase in social participation was associated with lower level of frailty (odds ratio: 0.96, 95% confidence interval: 0.93–0.99) and feeling lonely was associated with higher level of frailty (odds ratio: 2.90, 95% confidence interval: 1.44–5.84). Social isolation was not associated with frailty. Gender did not have moderating effect on these associations.

**Conclusions:**

This study observed that social isolation and loneliness had differential longitudinal association with level of frailty among community-dwelling older adults and suggested that loneliness and frailty should be measured and addressed concurrently among community-dwelling older adults.

**Supplementary Information:**

The online version contains supplementary material available at 10.1186/s12877-021-02745-2.

## Background

Social isolation and loneliness are two interrelated but empirically distinct concepts which reflect objective and subjective characteristics of social relationships, respectively [1]. As a structural indicator of social connection [2], social isolation reflects the extent of objective absence of or deficit in social connections and lack of social engagement [3]. As a functional indicator of social connection [2], loneliness is defined as “a distressing feeling that accompanies the perception that one’s social needs are not being met by the quantity or especially the quality of one’s social relationships.” [4] Evidence suggests that people around the world are more socially isolated nowadays than ever before [5] and older adults are generally at increased risk for social isolation and loneliness because they are more likely to experience many of the risk factors that can cause or exacerbate social isolation or loneliness, such as living alone, loss of loved ones, suffering from chronic illnesses, declined mobility, and vision or hearing impairment [6, 7]. In Singapore, population ageing, change in family structure, and shift towards nuclear families increase the likelihood of social isolation [8] and loneliness. Social isolation and loneliness have been recognized as emerging global public health issues that cast significant and growing influence on a wide array of adverse health outcomes, spanning from physical and psychological conditions [9, 10] to mortality [11].

Frailty is a common geriatric syndrome characterized by cumulative presentation of clinically identifiable somatic deficits, decreased physiological reserve, and heightened vulnerability to stressors [12, 13]. Despite no consensus definition of frailty or operational criteria for frailty assessment, prior research has consistently shown that frail older adults are at increased risk of falls and disability [14, 15], hospitalisation [16, 17], and mortality [18, 19]. With the increase in the ageing population worldwide, the number of frail individuals has been rising steadily over the years, resulting in an increasing and substantial burden on health care and social care systems. Frailty has been identified as a strong driver of healthcare utilisation and costs both locally [20] and internationally [21]. To forge a frailty-ready community to meet the challenges of population ageing, a better understanding of the risk factors of frailty and identifying effective approaches to attenuate the development and progression or even reversion of frailty is essential.

Prior research has suggested that the association of social isolation and loneliness with frailty is bi-directional with the exact causal mechanism uncertain: while frailty gives rise to greater social isolation and loneliness [22, 23], social isolation and loneliness may also lead to increased risk of frailty [22, 24, 25]. Although the association between loneliness and frailty was consistently reported by both cross-sectional and prospective studies globally [23, 25, 26], there is a lack of consensus about the relationship between social isolation and frailty. A cross-sectional study in a sample of elderly Chinese population aged 70 and older suggested social isolation was associated with increasing frailty as measured by frailty index [27]. However, data from the Mexican older adults of the same age group showed that frailty was not associated with social isolation [28]. A study examining the association between frailty and social isolation both cross-sectionally and prospectively showed that frailty was only cross-sectionally associated with a smaller social network [23]. A few recent prospective studies [22, 29, 30] provided additional evidence on the relationship between social isolation and frailty by predicting future frailty using the baseline social contacts or social participation. A limitation of these cross-sectional and prospective studies is that an individual’s social isolation and loneliness as well as other time-varying covariates were treated as static, and their dynamic nature was ignored. As such, it remains unclear how changes in social isolation and loneliness over time are associated with change in level of frailty when accounting for other time-varying covariates. Hence, this study was conducted to assess the longitudinal associations between social isolation, loneliness, and level of frailty in community-dwelling older adults using panel data, and examine whether social isolation and loneliness had differential association with frailty. As previous research observed gender difference in association between social isolation and frailty [27], it is possible that gender might moderate the association between social isolation or loneliness and frailty. As such, the second objective of the study was to explore the moderating effect of gender on the associations.

## Methods

### Study design and participants

The panel data of this study was derived from the longitudinal Population Health Index (PHI) Survey, which was a representative cohort study of community-dwelling adults aged 21 years and above in the Central region of Singapore. The PHI survey was initiated in November 2015 and the subsequent data collection was conducted yearly in 2016/2017 and 2017/2018 using face-to-face interviews and interviewer-administrated questionnaires. The sampling design, participant recruitment and follow-up processes were detailed elsewhere [31] and the contents of the questionnaire were described in a previous study [32]. The PHI study was approved by the ethics review committee of the National Healthcare Group Domain Specific Review Board (Reference Number: 2015/00269) and was conducted in accordance with the Declaration of Helsinki. Written informed consent was obtained from all individual participants after they were being informed about the study objectives and the safeguards put in place so that confidentiality of the collected data is maintained.

A total of 685 participants from the longitudinal PHI survey were identified for this study based on the following two inclusion criteria: 1) aged 60 years and above at baseline, and 2) cognitively sound and responded to the survey independently. From these, we excluded participants with any missing responses for the variables of the model or only having one observation (*n* = 79). Finally, 606 participants were included in the data analysis. There were no significant differences in baseline socio-demographics, social connection indicators or frailty status among participants included and excluded from this study. Among these 606 participants included in the data analysis, 138 (22.8%) had two observations and 468 (77.2%) had three observations. In total, there were 1738 observations.

### Measures

#### Frailty

The level of frailty (CFS1-7) for each participant was determined using the Clinical Frailty Scale [33] and operationalised based on the information obtained from relevant questions included in the PHI survey questionnaire. These questions provided information on diagnosis of chronic conditions, dependency for any activities of daily living (ADLs), assistance required in any high-order instrumental activities of daily living (IADLs, including shopping, housekeeping, transportation, handing medication, and finances), whether often taking part in active recreation or regular fitness program, and presence of any active bothersome symptoms. An individual would be categorized as CFS7-Severely Frail if the individual 1) completely dependent for any ADLs; or 2) diagnosed with severe dementia; or 3) chair/bed bound. An individual would be categorized as either CFS6-Moderately Frail or CFS5-Mildly Frail based on the level of assistance required in any ADLs and 2) help required in number of high-order IADLs. A person having any diagnosed chronic diseases would be categorized as either CFS4-Vulnerable or CFS3-Managing Well depending on whether he/she had any active bothersome symptoms. A person having no diagnosed chronic diseases would be categorized as CFS1-Very Fit if he or she often took part in active recreation or regular fitness program, otherwise, the person would be categorized as CFS2-Well. The flowchart describing this process step-by-step was presented in the Figure A1 of a previous study [32].

#### Social isolation

Social isolation was measured by the two subscales of the 6-item Lubben Social Network Scale-6 (LSNS-6). The LSNS-6 is composed of a set of three questions that evaluate social connectedness with relatives (LSNS-6 Family subscale) and a comparable set of three questions that evaluate social connectedness with friends (LSNS-9 Friends subscale) [34]. Specifically, the questions of the LSNS-6 Family subscale are: “How many relatives do you see or hear from at least once a month?”, “How many relatives do you feel close to such that you could call on them for help?”, and “How many relatives do you feel at ease with that you can talk about private matters?”. The word “relatives” in these three questions are replaced with the word “friends” for the questions of the LSNS-6 Friends subscale. Each item has 6 response options: 0 = none, 1 = one, 2 = two, 3 = three or four, 4 = five through eight, 5 = nine or more. The LSNS-6 Family and LSNS-6 Friends subscale scores were derived by adding up the 3 items evaluating social connection with relatives and friends, respectively. Each subscale score ranges from 0 to 15, with lower score indicating greater isolation. The two subscales in the present study demonstrated good internal consistency reliability with Cronbach's alpha of 0.81 for family subscale and 0.80 for friend subscale.

#### Social participation

Social participation was measured by self-reporting using the social role domain of the Late-Life Function and Disability Instrument (Late-Life FDI) [35]. It contains nine items that reflect the frequency of performing various social and community tasks or activities including keeping in touch with others, visiting friends and family in their homes, providing care or assistance to others, working at a volunteer job, taking part in active recreation, travel out of town, inviting people into your home, going out with others to public places, and taking part in organized social activities. These frequency questions are phrased “How often do you (do a particular task)?” with five response options: 5 = Very often, 4 = Often, 3 = Once in a while, 2 = Almost never, and 1 = Never. Following the score table presented in the Late-Life FDI Manual [36], each raw summary score of the nine items was transformed to a scaled score ranging from 0 to 100 based on a Rasch model with 100 indicating better social participation or engagement and 0 indicating worse social participation. The social role domain of Late-Life FDI in the study demonstrated acceptable internal consistency reliability (Cronbach's alphas = 0.76).

#### Loneliness

Loneliness was assessed using the UCLA Loneliness Scale [37]. Each participant was asked the following three questions: “How often do you feel that you lack companionship?”, “How often do you feel left out?” and “How often do you feel isolated from others?”. Each question had three options to reflect the frequency:1 = Hardly ever, 2 = Some of the time, and 3 = Often). The values for each question were summed to get a loneliness score ranging from 3 to 9, with higher values indicating greater loneliness. Each participant was then categorized into three categories: “not long” (scored 3), “somewhat lonely” (scored 4–5), and “lonely” (scored 6–9). The scale has good internal reliability in this study with Cronbach's alpha = 0.87.

#### Covariates

Several time-varying variables were included as covariates to account for any potential confounding effects. These variables included socio-demographics including age, marital status (married/cohabiting vs single/divorced/widowed), employment status (employed vs unemployed), living arrangement (alone vs with others), and financial status assessed by self-reported money insufficiency for basic daily living (perceived money sufficiency vs insufficiency); lifestyle and health-related factors including current smoking status, alcohol misuse assessed by the Alcohol Use Disorders Identification Test Consumption screening tool [38], number of diagnosed chronic conditions (self-reported), number of long-term medications (< 3 medications vs ≥ 3 medications), functional independence measured by the Modified Barthel Index for ADL (score range: 0—100), and nutritional status (normal vs undernutrition) assessed by Mini Nutritional Assessment [39].

#### Statistical analysis

Baseline characteristics of the study participants were described using mean and standard deviation (SD) for continuous variables, and frequency and percentages for categorical variables.

Fixed-effects ordinal logistic regression for panel data was conducted to examine the associations of change in social isolation, social participation, and loneliness with change in level of frailty over time. This approach has several strengths. Firstly, fixed-effects model considers dynamic relationships between independent and dependent variables. This is especially helpful when exploring the associations of factors such as social isolation, social participation, and loneliness, and outcomes such as level of frailty which are likely to change dynamically over time and are likely to be influenced by other time-varying covariates. Secondly, fixed-effects models explore within-person variation by taking individuals as their own reference point over time. As such, fixed-effects regression accounts for all time-invariant factors (e.g. gender, ethnicity, and the highest education attained) and their heterogeneity even if unobserved [40]. Thirdly, fixed-effects models relax the distributional and independence assumptions on individual-specific error terms, which makes them useful for the estimation of causal effects as it accounts for any potential endogeneity stemming from individual’s time-invariant characteristics [40]. Random-effects models were also conducted to assess the panel structure of the data and Hausman tests were used to confirm the selection of fixed-effects models over random-effects models if *p* < 0.05.

Three fixed-effects ordinal logistic regression models were run to examine the associations of social isolation and social participation (continuous variables) and loneliness status (not lonely vs lonely) with level of frailty (a seven-level ordered variable): Model 1 included social isolation, social participation, and loneliness status adjusted for all time-invariant factors (e.g., gender, ethnicity, and the highest education attained). Model 2 additionally adjusted for time-varying demographic factors including age, marital status, employment status and living arrangement. Model 3 additionally adjusted for lifestyle and health-related factors including current smoking status, alcohol misuse, number of chronic conditions, number of long-term medications, current nutritional status, and functional independence. To examine the moderating effect of gender on the associations between social isolation, social participation, and loneliness and level of frailty, the interaction terms of gender and each social connection indicators were added to the Model 3. Odds ratios (ORs) and 95% confidence intervals (CIs) were reported for each model.

We conducted sensitivity analyses (SAs) based on the Model 3 using 1) isolation status (not isolated vs isolated) instead of isolation score as the independent variable (SA 1), and 2) loneliness score instead of loneliness status as the independent variable (SA 2). We also ran a fixed-effects logistic regression on the panel data using dichotomous frailty (non-frail: CFS1-3 vs. frail: CFS4-7) as the outcome variable, controlling for all the covariates included in the Model 3 (SA 3). Furthermore, as LSNS-6 Family, LSNS-6 Friends, and social participation were moderately associated with each other (*r* = 0.42–0.53, *p* < 0.001) and mildly associated with loneliness (*r* = -0.22 to -0.28, *p* < 0.001), we also conducted sensitivity analyses to examine the individual association of social isolation, social participation and loneliness with level of frailty by only including one social connection indicator in the model and adjusted for all the time-vary factors included in the Model 3 (SA 4a-4d).

All the analyses were carried out using Stata/SE 16.1 for Windows (StataCorp, College Station, TX) and a p value of 0.05 was set as the level of significance for all tests.

## Results

### Study participants

The baseline characteristics of the participants are described in Table 1. Of the 606 participants, 57.6% were females, 84.3% were Chinese and 52.0% had no formal education or primary school qualification only. At baseline, the mean age of the participants was 70.9 years old, 58.6% were married, and 46.0% were out of labour force (inactive).

**Table 1:** Characteristics of participants at baseline (*N* = 606)

Characteristics	n / mean	% /SD
***Time-invariant characteristics***		
**Female**	349	57.6
**Ethnicity**		
Chinese	511	84.3
Malay	31	5.1
Indian	56	9.2
Others	8	1.3
**Highest education attended**		
No formal education	207	34.2
Primary school	108	17.8
Secondary school	217	35.8
Post-secondary school & above	74	12.2
***Time-variant characteristics***		
**Age (Mean, SD)**	*70.1*	*7.9*
**marital status**		
Married	355	58.6
Single/divorced/widowed	251	41.4
**Employment status**		
Employed	185	30.5
Unemployed	142	23.4
Inactive	279	46.0
**Living alone**	119	19.6
**Self-reported money insufficiency**	103	17.0
**Currently smoking**	54	8.9
**Alcohol misuse**	69	11.4
**Number of chronic conditions (Mean, SD)**	*2.7*	*2.0*
**Number of medications**		
0	171	28.2
1 or 2	208	34.3
3 or more	227	37.5
**Nutritional status: undernutrition**	80	13.2
**Functional independence- MBI score (Mean, SD)**	*97.8*	*9.3*
**LSNS-6 Family (range 0–15) (Mean, SD)**	*8.4*	*3.5*
Isolated from relatives (LSNS-6 Family < 6)	107	17.7
**LSNS-6 Friends (range 0–15) (Mean, SD)**	*6.0*	*4.0*
Isolated from friends (LSNS-6 Friends < 6)	287	47.4
**Social participation (range 0–100) (Mean, SD)**	*39.4*	*9.4*
**Loneliness (range3-9) (Mean, SD)**	*3.4*	*1.0*
Not lonely	636	93.0
Lonely (loneliness score ≥ 6)	44	7.3

*SD standard deviation, MBI Modified Barthel Index*

At baseline, 19.6% were living alone, 17.7% of participants were categorized as “isolated” based on the LSNS-6 Family subscale and 47.4% were categorized as “isolated” based on the LSNS-6 Friends subscale. There were 7.3% individuals categorized as “lonely” (loneliness score < 6). The descriptive summary of social isolation, social participation, loneliness, and level of frailty for participants at each time point is presented in Supplementary Tables 1a and 1b (Additional File 1).

### Association of social isolation, social participation, and loneliness with level of frailty

The results of the fixed-effects models are presented in Table 2 and the results of the random-effects models and Hausman test are presented in Supplementary Table 2 (Additional File 1). As suggested by Hausman test results (all *p*-values < 0.05), we reported the results of the fixed-effects models.

**Table 2:** Associations of social isolation, social participation, and loneliness with level of frailty (Fixed-effects models)

	**Model 1**		**Model 2**		**Model 3**	
	**OR (95%CI)**	***p*-value**	**OR (95%CI)**	***p*-value**	**OR (95%CI)**	***p*-value**
LSNS-6 Family	1.04 (0.97—1.11)	0.255	1.04 (0.97—1.11)	0.250	1.05 (0.97—1.14)	0.231
LSNS-6 Friends	0.99 (0.93—1.06)	0.848	1.00 (0.94—1.07)	0.991	0.99 (0.92—1.07)	0.782
Social participation	0.95 (0.93—0.98)	< 0.001	0.95 (0.93—0.98)	< 0.001	0.96 (0.93—0.99)	0.019
Lonely (Ref: Not lonely)	2.43 (1.17—5.04)	0.017	2.62 (1.24—5.52)	0.011	2.90 (1.44—5.84)	0.003
Age			1.15 (0.97—1.35)	0.105	0.89 (0.74—1.08)	0.233
Marital status (Ref: Married)						
Single/divorced/widowed			1.34 (0.43—4.13)	0.616	1.29 (0.13—12.40)	0.827
Employment status (Ref: Employed)						
Unemployed			0.42 (0.21—0.84)	0.013	0.37 (0.18—0.74)	0.005
Inactive			0.75 (0.38—1.45)	0.386	0.48 (0.23—1.00)	0.050
Living alone (Ref: Living with others)			0.58 (0.27—1.28)	0.177	0.42 (0.17—1.07)	0.070
Self-reported money insufficiency (Ref: Sufficient)			1.35 (0.83—2.21)	0.224	1.54 (0.87—2.75)	0.142
Currently smoking (Ref: Not smoking)					0.79 (0.14 – 4.33)	0.782
Alcohol misuse (Ref: No misuse)					0.94 (0.41 – 2.16)	0.885
Number of chronic conditions					3.48 (1.92 – 6.31)	< 0.001
Number of medications (Ref: 0–2)						
3 or more					1.99 (1.01 – 3.89)	0.045
Nutritional status (Ref: Normal)						
Undernutrition					1.80 (0.93—3.48)	0.083
Functional independence					0.76 (0.67 – 0.86)	< 0.001

*Number of observations: 782; number of individuals: 282. Model 1 accounted for all time-invariant factors. Model2 additionally adjusted for time-variant demographic factors including age, marital status, employment status and living arrangement. Model 3 additionally adjusted for lifestyle and health-related factors including current smoking status, alcohol misuse, number of chronic conditions, number of long-term medications, current nutritional status, and functional independence. OR: odds ratio; 95%CI: 95% confidence interval*

As shown in Table 2, increased frequency of social participation was consistently associated with lower level of frailty with OR of 0.95 remaining unchanged in Model 1 and Model 2 and 0.96 in Model 3 (Table 2). However, no significant association between either LSNS-6 Family or LSNS-6 Friends and level of frailty was observed in any model.

Feeling lonely was consistently associated with higher level of frailty (Model1: OR = 2.43, Model 2: OR = 2.62, Model 3: OR = 2.90, all *p* < 0.05), and the association increased slightly after adjusting for time-varying socio-demographic and health-related factors.

### The moderating effect of gender.

After adding the interaction terms of gender and each social connection indicator to Model 3, while feeling lonely remained significantly associated with level of frailty, the association between social participation and level of frailty attenuated to be non-significant. None of the interaction terms showed association with level of frailty (Table 3, all *p*-values > 0.05).

**Table 3:** Associations of social isolation, social participation, and loneliness with level of frailty with interaction terms

Social connection indicator	OR (95%CI)	*p*-value
LSNS-6 Family	1.03 (0.91—1.17)	0.614
LSNS-6 Family#female	1.03 (0.87—1.23)	0.697
LSNS-6 Friends	0.99 (0.89—1.11)	0.904
LSNS-6 Friends#female	0.99 (0.85—1.15)	0.863
Social participation	0.96 (0.91 – 1.00)	0.065
Social participation#female	1.01 (0.95—1.08)	0.642
Lonely (Ref: Not lonely)	1.39 (1.02—1.89)	0.036
Lonely (Ref: Not lonely)#female	0.80 (0.53—1.21)	0.294

*Number of observations: 782; number of individuals: 282. Adjusted for all time-invariant factors; time-variant demographic factors including age, marital status, employment status and living arrangement lifestyle and health-related factors including current smoking status, alcohol misuse, number of chronic conditions, number of long-term medications, current nutritional status, and functional independence. OR: odds ratio; 95%CI: 95% confidence interval*

### Sensitivity analyses

The SA 1 results in Supplementary Table 3a (Additional File 1) showed that when the two LSNS-6 subscale scores were replaced with the dichotomous social isolation status (not isolated vs isolated) in Model 3, the significant association of social participation (OR: 0.96, *p* = 0.015) and feeling lonely (OR: 3.13, *p* = 0.001) with level of frailty remained. When loneliness score was replaced with the dichotomous loneliness status (not lonely vs lonely) in the Model 3 (SA 2 in Supplementary Table 3b), social participation (OR: 0.96, *p* = 0.021) and feeling lonely (OR: 1.25, *p* = 0.029) were still significantly associated with level of frailty.

When using dichotomous frailty (not frailty vs frailty) as the outcome (SA 3 in Supplementary Table 4), the association between social participation and feeling lonely and frailty remained: older adults with more frequent social participation was associated with lower odds of frailty (OR: 0.92, *p* < 0.001) and those reported lonely had higher odds of frailty (OR: 3.61, *p* = 0.002). Furthermore, LSNS-6 Family was also associated with higher odds of frailty (OR: 1.11, *p* = 0.005).

Examining the individual social connection indicators’ association with level of frailty by including only one indicator in the full adjusted model (SA4a-d) did not show any material changes to the findings (Supplementary Table 5).

## Discussion

This was the first study to examine the association of both objective and subjective social connection with level of frailty among community-dwelling older adults using panel data from a longitudinal survey in Singapore. Frequency of social participation and loneliness were found to be independently associated with level of frailty and their associations were independent of time-invariant factors such as gender, ethnicity, and highest education attained, and independent of changes in socio-demographic factors, lifestyle and health-related factors which were identified in previous studies [41, 42].

Existing literature on association between social connections and frailty used different tools to assess social isolation, social participation, and frailty. This variability may contribute to the variations in observed associations, leading to the incomplete understanding of the association between social connections (including social isolation, social participation, and loneliness) and frailty [43]. Prior literature suggested the potential bi-directionality of their association [25, 44], however, as social isolation and loneliness are recognized as modifiable social determinants of health [7], they were more commonly used as independent variables and frailty was used as the dependent variable. This study chose frailty as the dependent variable based on current local efforts in building a frailty-friendly community in Singapore. We used CFS instead of Fried phenotype or Frailty Index [22, 30] to assess frailty as it is widely used in our local community for frailty screening.

Unlike recent longitudinal studies [22, 45] which found social isolation was associated with frailty measured using Frailty Index, our study did not observe any association between changes in social isolation (either social isolation from relatives or from friends) and changes in level of frailty over time. It is unclear whether the inconsistent finding was attributed to the use of different frailty and / or social isolation tool. Although different social participation tools were used, our findings were consistent with the findings of previous studies [22, 24, 28, 30] and found that increase in social participation was associated with decrease in level of frailty, and this association was independent of living arrangement and social isolation.

Similar to the findings from existing evidence [24–26, 45], our study showed that feeling lonely was consistently associated with higher odds of worsened frailty and their association was independent of social isolation and social participation. The prevalence of loneliness among the study population (7.3% for those with loneliness score ≥ 6, and 16.8% for those with loneliness score > 3) was relatively lower than the prevalence (23%—24%) reported by three local studies [8, 46, 47] which used different approaches for sampling or scale scoring. Besides, the differences in the profile of older adults and changes in the community over the past years may also lead to the decline in the prevalence of loneliness in Singapore: at individual level, older adults nowadays stay in the labour force for a longer period, and they are more educated and more likely to seek ways to curb feeling of loneliness such as using of social media platforms; while at community level, various befriending programmes/services or community programmes and support make seniors feel less excluded [48].

The mechanisms underlying the differential association between social isolation, social participation, loneliness, and frailty among older adults are likely complex. Although social isolation was found to be a risk factor for loneliness in older adults [49], social isolation and loneliness are distinct concepts. While being isolated for a short period could be by choice and has less impact on health; loneliness, as a negative emotional feeling, may contribute to a series of physical and mental health issues including cardiovascular disease and stroke, increased stress and depression, and cognitive decline [50, 51]; thereby increasing the risk of development and progression of frailty. Social participation among older adults directly increases social interactions which has the potential to reduce cognitive decline, lowers the risk of depression, and creates a sense of belonging which alleviate feeling of loneliness [52]. Furthermore, social participation also increases physical activities, which reduces the risk of frailty.

While many studies reported gender differences in social isolation [53], loneliness [54, 55] and frailty [56, 57], our study did not observe significant association between the interaction term of gender and any social indicators and frailty, suggesting that gender did not moderate the association between either changes in social isolation, social participation, or loneliness and level of frailty. Although a few cross-sectional and prospective studies found that many socio-demographic characteristics such as marital status and living arrangement were associated with cross-sectional and/or future frailty [42, 58], our study did not observe any association between change in these characteristics and frailty during the study period.

The association of social participation and loneliness with frailty implies that promoting social participation or engagement (both frequency and types) and addressing loneliness may contribute to prevention of the development of frailty or attenuation of the frailty progression. Considering the association between social participation and frailty might be bidirectional, more attention should be paid to the potential risk of loneliness among frail individuals, and it is worth exploring strategies or interventions to engage frail individuals in social activities in the community.

The major strength of the study was the use of panel data fixed-effects regressions that account for time-varying covariates. Although it is still possible that some factors that may influence social indicators and frailty were not accounted for, by using each participant as their own control, the influence of time-invariant factors was eliminated [59] and the associations identified reflect how change in social connection indicators dynamically influenced level of frailty. Secondly, we examined multiple indicators of social connection (social isolation from family and friends, social participation, and loneliness) and conducted sensitivity analyses with each indicator in one model. This gives a relatively detailed description of one’s social connection and enables us to examine the link between each specific component of social connection and frailty. However, due to the nature of observational studies, we could not infer any causality, especially that some potential time-varying covariates that might cause frailty (e.g., fracture, some acute conditions) were not observed. And it is still possible that deterioration in physical or mental functioning caused the reduction in social participation and loneliness. In additional, the data analysed was collected at yearly basis with an attrition rate of 11.8% and 17.8% for the first and second follow-up respectively. This may result in attrition bias and underestimation of the associations.

## Conclusions

This study observed that social isolation, social participation, and loneliness had differential longitudinal association with level of frailty among community-dwelling older adults in Singapore. Social participation and feeling of loneliness were independently associated with higher level of frailty in older adults and gender did not moderate the associations. Our findings suggest that loneliness and frailty should be measured and addressed concurrently among community-dwelling older adults.

## Supplementary Information

**Additional file 1:** **Table 1a. **The descriptive summary of social connection indicators across three time points. **Table 1b. **The distribution of level of frailty across three time points. **Table 2. **Associations of social isolation, social participation, and loneliness with level of frailty (Random-effects models) and Hausman tests. **Table 3a. **Association of social isolation status (dichotomous), social participation, and loneliness status (dichotomous) with level of frailty (Sensitivity analysis 1). **Table 3b. **Association of social isolation (continuous), social participation, and loneliness score (continuous) with level of frailty (Sensitivity analysis 2). **Table 4. **Association of social isolation, social participation, and loneliness status with frailty (dichotomous) based on Random-effects model (Sensitivity analysis 3). **Table 5. **Association of social connectedness and loneliness score with level of frailty, each social indicator entered separately into each model (Sensitivity analysis 4).

## Data Availability

The datasets used and/or analysed during the current study are available from the corresponding author on reasonable request.

## Abbreviations

ADLs: Activities of daily living
MBI: Modified Barthel Index
CFS: Clinical Frailty Scale
CI: Confidence interval
IADLs: Instrumental activities of daily living
LSNS-6: 6-Item Lubben Social Network Scale-6
Late-Life FDI: Late-Life Function and Disability Instrument
OR: Odds ratio
PHI: Population Health Index
SD: Standard deviation
