# Supplementary material for: Associations of social isolation, social participation, and loneliness with frailty in older adults in Singapore: a panel data analysis
Source: BMC Geriatr. 2022 Jan 6;22:26. doi: 10.1186/s12877-021-02745-2 (PMC8734362; doi:10.1186/s12877-021-02745-2)
Supplement: Supplementary file 1 — Additional file 1: Table 1a. The descriptive summary of social connection indicators across three time points. Table 1b. The distribution of level of frailty across three time points. Table 2. Associations of social isolation, social participation, and loneliness with level of frailty (Random-effects models) and Hausman tests. Table 3a. Association of social isolation status (dichotomous), social participation, and loneliness status (dichotomous) with level of frailty (Sensitivity analysis 1). Table 3b. Association of social isolation (continuous), social participation, and loneliness score (continuous) with level of frailty (Sensitivity analysis 2). Table 4. Association of social isolation, social participation, and loneliness status with frailty (dichotomous) based on Random-effects model (Sensitivity analysis 3). Table 5. Association of social connectedness and loneliness score with level of frailty, each social indicator entered separately into each model (Sensitivity analysis 4). [file 12877_2021_2745_MOESM1_ESM.docx]

**Title page**

**Title:** Associations of social isolation, social participation, and loneliness with frailty in older adults in Singapore: a panel data analysis

**Authors:** Lixia Ge^1^, Chun Wei Yap^1^, Bee Hoon Heng^1^

^1^ Health Services & Outcomes Research, National Healthcare Group, Singapore

Supplementary Table 1a: The descriptive summary of social connection indicators across three time points

| **Social connection indicator** | **Time point** | **N** | **Mean (SD)** | **Median (IQR)** | **n (%)** |
| --- | --- | --- | --- | --- | --- |
| LSNS-6 Family | Baseline | 606 | 8.4 (3.5) | 9 (6 - 11) | 107 (17.7) |
|  | 1-year followup | 599 | 8.8 (3.6) | 9 (6 - 11) | 111 (18.5) |
|  | 2-year followup | 533 | 7.9 (3.4) | 8 (5 - 10) | 134 (25.1) |
| LSNS-6 Friends | Baseline | 606 | 6.0 (4.0) | 6 (3 - 9) | 287 (47.4) |
|  | 1-year followup | 599 | 6.2 (4.1) | 6 (3 - 9) | 286 (47.8) |
|  | 2-year followup | 533 | 6.2 (3.9) | 6 (3 - 9) | 242 (45.4) |
| Social participation | Baseline | 606 | 39.4 (9.4) | 39.9 (34.7 - 44.9) |  |
|  | 1-year followup | 599 | 40.2 (9.0) | 41.1 (34.7 - 46.3) |  |
|  | 2-year followup | 533 | 39.1 (10.1) | 39.9 (34.7 - 44.9) |  |
| Loneliness score /Feeling lonely | Baseline | 606 | 3.4 (1.0) | 3 (3 - 3) | 44 (7.3) |
|  | 1-year followup | 599 | 3.4 (1.1) | 3 (3 - 3) | 49 (8.2) |
|  | 2-year followup | 533 | 3.3 (0.8) | 3 (3 - 3) | 23 (4.3) |
| ADL score | Baseline | 606 | 97.8 (9.3) | 100 (100 - 100) |  |
|  | 1-year followup | 599 | 96.6 (12.7) | 100 (100 - 100) |  |
|  | 2-year followup | 533 | 98.2 (8.1) | 100 (100 - 100) |  |

Supplementary Table 1b: The distribution of level of frailty across three time points

| **Level of frailty** | **Baseline (n=606)** | **1-year followup (n=599)** | **2-year followup (n=533)** |
| --- | --- | --- | --- |
| Very fit | 22 (3.6) | 24 (4.0) | 32 (6.0) |
| Well | 141 (23.3) | 135 (22.5) | 115 (21.6) |
| Managing well | 330 (54.5) | 305 (50.9) | 287 (53.9) |
| Vulnerable | 49 (8.1) | 58 (9.7) | 42 (7.9) |
| Mildly frail | 33 (5.5) | 43 (7.2) | 40 (7.5) |
| Moderately frail | 21 (3.5) | 20 (3.3) | 13 (2.4) |
| Severely frail | 10 (1.7) | 14 (2.3) | 4 (0.8) |

Supplementary Table 2: Associations of social isolation, social participation, and loneliness with level of frailty (Random-effects models) and Hausman tests

|  | **Model 1** | | **Model2** | | **Model 3** | |
| --- | --- | --- | --- | --- | --- | --- |
|  | **OR (95%CI)** | **p-value** | **OR (95%CI)** | **p-value** | **OR (95%CI)** | **p-value** |
| LSNS-6 Family | 1.07 (1.02 - 1.13) | 0.006 | 1.06 (1.01 - 1.11) | 0.019 | 1.06 (1.01 - 1.11) | 0.020 |
| LSNS-6 Friends | 0.94 (0.90 - 0.99) | 0.019 | 0.96 (0.91 – 1.00) | 0.070 | 0.99 (0.95 - 1.03) | 0.641 |
| Social participation | 0.92 (0.90 - 0.94) | <0.001 | 0.93 (0.91 - 0.95) | <0.001 | 0.96 (0.94 - 0.98) | <0.001 |
| Lonely (Ref: Not lonely) | 4.42 (2.52 - 7.76) | <0.001 | 4.11 (2.35 - 7.18) | <0.001 | 2.15 (1.24 - 3.76) | 0.007 |
| Female (Ref: Male) | 1.15 (0.68 - 1.95) | 0.592 | 2.04 (1.22 - 3.41) | 0.007 | 1.90 (1.25 - 2.89) | 0.003 |
| Non-Chinese (Ref: Chinese) | 3.23 (1.58 - 6.61) | <0.001 | 3.15 (1.65 - 6.03) | <0.001 | 1.13 (0.69 - 1.85) | 0.637 |
| Highest education attended (Ref: No formal education) |  |  |  |  |  |  |
| Primary school | 0.30 (0.14 - 0.64) | 0.002 | 0.79 (0.39 - 1.6) | 0.51 | 0.76 (0.44 - 1.30) | 0.316 |
| Secondary school | 0.21 (0.11 - 0.40) | <0.001 | 0.65 (0.35 - 1.19) | 0.16 | 0.87 (0.55 - 1.38) | 0.557 |
| Post-secondary school & above | 0.51 (0.21 - 1.25) | 0.141 | 1.56 (0.68 - 3.60) | 0.295 | 1.57 (0.82 - 2.98) | 0.174 |
| Age |  |  | 1.20 (1.16 - 1.24) | <0.001 | 1.10 (1.07 - 1.13) | <0.001 |
| Marital status (Ref: Married) |  |  | 0.83 (0.50 - 1.37) | 0.463 | 0.77 (0.51 - 1.16) | 0.213 |
| Employment status (Ref: Employed) |  |  |  |  |  |  |
| Unemployed |  |  | 0.83 (0.50 - 1.39) | 0.476 | 0.93 (0.59 - 1.46) | 0.752 |
| Inactive |  |  | 1.46 (0.92 - 2.32) | 0.111 | 0.94 (0.63 - 1.40) | 0.749 |
| Living alone (Ref: Living with others) |  |  | 0.46 (0.28 - 0.77) | 0.003 | 0.65 (0.41 - 1.02) | 0.062 |
| Self-reported money insufficiency (Ref: Sufficient) |  |  | 1.48 (0.99 - 2.22) | 0.054 | 1.33 (0.91 - 1.96) | 0.143 |
| Currently smoking (Ref: Not smoking) |  |  |  |  | 0.94 (0.50 - 1.77) | 0.842 |
| Alcohol misuse (Ref: No misuse) |  |  |  |  | 1.31 (0.81 - 2.12) | 0.271 |
| Number of chronic conditions |  |  |  |  | 2.34 (2.07 - 2.64) | <0.001 |
| Number of medications (Ref: 0-2) |  |  |  |  |  |  |
| 3 or more |  |  |  |  | 2.32 (1.58 - 3.40) | <0.001 |
| Nutritional status (Ref: Normal) |  |  |  |  |  |  |
| Undernutrition |  |  |  |  | 1.97 (1.28 - 3.04) | 0.002 |
| Functional independence |  |  |  |  | 0.85 (0.82 - 0.87) | <0.001 |
| *Hausman test* |  | *<0.001* |  | *<0.001* |  | *0.014* |

*Model 1 adjusted for time-invariant factors including gender, non-Chinese, and highest education attended. Model2 additionally adjusted for time-variant demographic factors including age, marital status, employment status and living arrangement. Model 3 additionally adjusted for* *lifestyle and health-related factors including current smoking status,* *alcohol misuse, number of chronic conditions, number of long-term medications current nutritional status, and functional independence.* *OR: odds ratio; 95%CI: 95% confidence interval.*

Supplementary Table 3a: Association of social isolation status (dichotomous), social participation, and loneliness status (dichotomous) with level of frailty (Sensitivity analysis 1)

| **Social connection indicator** | **OR (95%CI)** | **p-value** |
| --- | --- | --- |
| Isolated from family  (Ref: not isolated from relatives) | 0.52 (0.28 - 0.96) | 0.037 |
| Isolated from friends  (Ref: not isolated from friends) | 1.02 (0.63 - 1.64) | 0.946 |
| Social participation | 0.96 (0.93 - 0.99) | 0.015 |
| Lonely (Ref: not lonely) | 3.13 (1.55 - 6.32) | 0.001 |

*Adjusted for all time-invariant factors; time-variant demographic factors including age, marital status, employment status and living arrangement; lifestyle and health-related factors including current smoking status, alcohol misuse, number of chronic conditions, number of long-term medications, current nutritional status, and functional independence.* *OR: odds ratio; 95%CI: 95% confidence interval.*

Supplementary Table 3b: Association of social isolation (continuous), social participation, and loneliness score (continuous) with level of frailty (Sensitivity analysis 2)

| **Social connection indicator** | **OR (95%CI)** | **p-value** |
| --- | --- | --- |
| LSNS-6 Family | 1.05 (0.97 - 1.14) | 0.244 |
| LSNS-6 Friends | 0.99 (0.91 - 1.07) | 0.741 |
| Social participation | 0.96 (0.93 - 0.99) | 0.021 |
| Loneliness score | 1.25 (1.02 - 1.52) | 0.029 |

*Adjusted for all time-invariant factors; time-variant demographic factors including age, marital status, employment status and living arrangement; lifestyle and health-related factors including current smoking status, alcohol misuse, number of chronic conditions, number of long-term medications, current nutritional status, and functional independence.* *OR: odds ratio; 95%CI: 95% confidence interval.*

Supplementary Table 4: Association of social isolation, social participation, and loneliness status with frailty (dichotomous) based on Random-effects model (Sensitivity analysis 3)

| **Social connection indicator** | **OR (95%CI)** | **p-value** |
| --- | --- | --- |
| LSNS-6 Family | 1.13 (1.03 - 1.20) | 0.005 |
| LSNS-6 Friends | 0.99 (0.92 - 1.06) | 0.689 |
| Social participation | 0.92 (0.89 - 0.96) | <0.001 |
| Lonely (Ref: Not lonely) | 3.61 (1.63 – 8.00) | 0.002 |

*Adjusted for time-invariant factors including gender, non-Chinese, highest education attained; time-variant demographic factors including age, marital status, employment status and living arrangement; lifestyle and health-related factors including current smoking status, alcohol misuse, number of chronic conditions, number of long-term medications, current nutritional status, and functional independence.* *OR: odds ratio; 95%CI: 95% confidence interval.*

Supplementary Table 5: Association of social connectedness and loneliness score with level of frailty, each social indicator entered separately into each model (Sensitivity analysis 4)

| **Social connection indicator** | **Odds Ratio (95%CI)** | **p-value** |
| --- | --- | --- |
| SA4a. LSNS-6 Family | 1.03 (0.96 - 1.11) | 0.363 |
| SA4b. LSNS-6 Friends | 0.98 (0.91 - 1.05) | 0.547 |
| SA4c. Social participation | 0.97 (0.94 – 1.00) | 0.031 |
| SA4d. Lonely (Ref: Not lonely) | 2.68 (1.32 - 5.44) | 0.007 |

*Adjusted for all time-invariant factors; time-variant demographic factors including age, marital status, employment status and living arrangement; and lifestyle and health-related factors including current smoking status, alcohol misuse, number of chronic conditions, number of long-term medications current nutritional status, and functional independence.* *OR: odds ratio; 95%CI: 95% confidence interval.*
